# Crosstalk between chemotherapeutics and tumor-associated macrophages (TAMs): implications for tumor response

**DOI:** 10.1007/s00432-026-06575-w

**Published:** 2026-08-04

**Authors:** Bożena Pezała, Magdalena Radziszewska, Ewa Augustin

**Affiliations:** 1 Department of Pharmaceutical Technology and Biochemistry, Faculty of Chemistry, Gdańsk University of Technology, Gabriela Narutowicza 11/12, 80-233 Gdańsk, Poland; 2 Department of Internal Medicine, Pulmonary Diseases and Allergy, Medical University of Warsaw, Banacha 1a, 02- 097 Warsaw, Poland

**Keywords:** Tumor-associated macrophages (TAMs), Tumor microenvironment (TME), Cancer, Chemotherapeutics

## Abstract

The understanding of cancer has evolved from a group of mutated cancerous cells to a complex ecosystem comprising many cellular and non-cellular components. Tumor-associated macrophages (TAMs) are one of the most abundant immune cells in the tumor microenvironment (TME). They actively contribute to cancer outcomes in two ways, either promoting its development and progression or stimulating immune response to combat tumor. Recently, TAMs have been recognized not only as prognostic markers for the outcome of anti-cancer therapy, but also as major players in shaping treatment response. It was also established that therapeutic agents may affect TAMs’ phenotype, and in consequence modulate tumor’s behavior. Therefore, reprogramming of TAMs in order to enhance tumor infiltration and overcome immune suppression is also currently a hot topic in preclinical research. In this review, we discuss the interactions between chemotherapeutic agents and tumor-associated macrophages and their potential consequences for tumor response.

## Introduction

Our understanding of cancer has evolved substantially over recent decades. Tumors are no longer viewed as mere collections of genetically transformed cells but rather as complex and dynamic ecosystems. Tumor microenvironment (TME) consists of various cellular and non-cellular components that interact with tumor cells and with each other. That interplay between TME and cancer strongly affects its development and progression. The TME is a highly structured, heterogeneous entity. Its composition varies between different cancer types, but it usually features immune cells (T-cells, natural killers, dendritic cells, macrophages), blood vessels, endothelial cells, fibroblasts, and extracellular matrix (Anderson and Simon 2020; de Visser and Joyce 2023).

The immune system plays a pivotal role in cancer development and progression. It is well known that chronic inflammation may initiate tumor development. For instance, infection with human papillomaviruses (HPVs) is responsible for more than 90% of cervical cancer cases. HPV-driven oncogenesis is primarily mediated by the viral oncoproteins E6 and E7, which promote degradation of p53 and inactivation of the retinoblastoma protein (pRB), leading to impaired apoptosis and deregulation of the cell cycle (Hoppe-Seyler et al. 2018). Moreover, E6 and E7 have been shown to activate the COX–prostaglandin pathway, a key regulator of inflammation, and to increase reactive oxygen species (ROS) production, which further amplifies inflammatory signaling and contributes to the progression of HPV-induced carcinogenesis (Hemmat and Bannazadeh Baghi 2019). On the other hand, acute inflammation may be used to combat tumor. Activation of innate and adaptive immune response by *Mycobacterium bovis* vaccine can eradicate bladder cancer (Mukherjee et al. 2021).

**Fig. 1:**
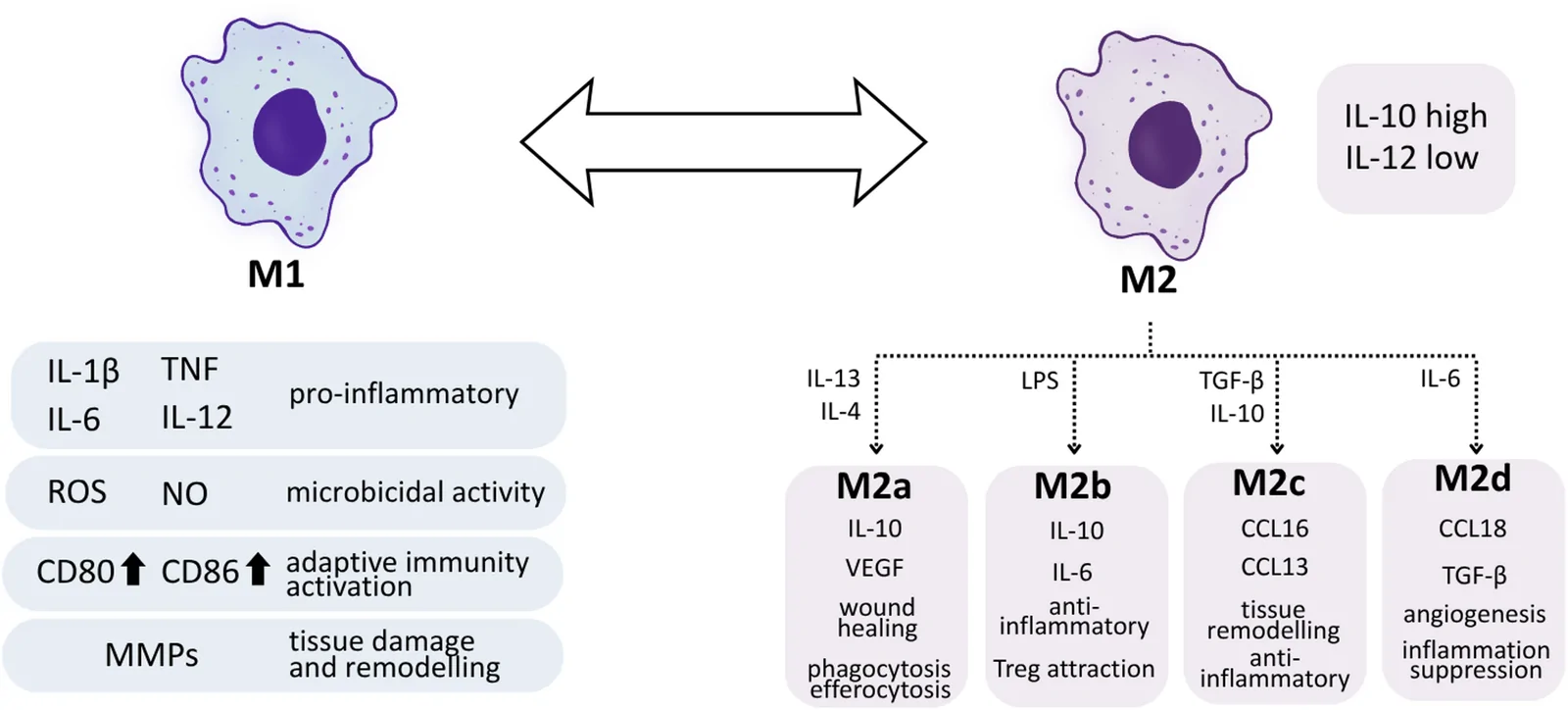
Macrophage phenotypes

The immune system not only plays a role in cancer development, but also modulates its behavior. One of the most prevalent in tumor immune cells, which actively modify cancer cell dynamics, are macrophages (Anderson and Simon 2020). Macrophages, key components of the innate immune system, exhibit remarkable plasticity, enabling them to adapt their phenotype in response to diverse stimuli and perform a wide range of functions. Although macrophages can adopt a wide spectrum of activation states (Fig. 1), they are commonly classified into two main subsets: classically activated M1 macrophages, which exhibit pro-inflammatory properties, and alternatively activated M2 macrophages, which are associated with anti-inflammatory functions (Baay et al. 2011; Gonzalez et al. 2018; Anderson and Simon 2020; de Visser and Joyce 2023; Yan et al. 2024). However, it is important to emphasize that the M1/M2 classification does not fully reflect the heterogeneity of macrophages. Therefore, as in this review, this simplified classification is used primarily to provide a framework for discussing this highly complex cell population.

M1 macrophages are activated by microbial products, such as LPS, or proinflammatory cytokines, such as IFN-γ, TNF or GM-CSF. Those M1 macrophages act as the main effector cells focused on immediate defense and pathogen clearance. They initiate pro-inflammatory response through secreting pro-inflammatory cytokines, such as TNF, IL-1β, IL-6, and IL-12. Their ability to generate reactive oxygen species (ROS) and nitric oxide (NO) – crucial molecules involved in the elimination of pathogens – accounts for their microbicidal activity. Upregulation of Major Histocompatibility Complex (MHC) class II molecules and costimulatory molecules (CD80, CD86) enables macrophages to present antigens to T cells, which in turn activate adaptive immunity. Additionally, they mediate tissue damage and remodeling through secreting ROS and Matrix Metalloproteinases (MMPs), which break down extracellular matrix components (Strizova et al. 2023; Yan et al. 2024; Deng et al. 2025).

M2 macrophages balance pro-inflammatory M1 macrophages to maintain homeostasis. They are key players in inflammatory response suppression, wound healing, and tissue repair. Even though all M2 macrophages are characterized by high levels of IL-10 and low levels of IL-12, they can be classified into four main subtypes – M2a, M2b, M2c, and M2d. M2a are activated by IL-4 and IL-13. They secrete, among others, IL-10 and VEGF and play a crucial role in tissue remodeling and wound healing, as well as, through phagocytosis and efferocytosis, in pathogen and apoptotic cell clearance. M2b are induced by TLR agonists, such as LPS, and immune complexes. By secreting high levels of anti-inflammatory IL-6, IL-10, and CCL1, they create an immunosuppressive environment and attract Treg (T regulatory) cells. M2c phenotype occurs upon TGF-β, IL-10, or glucocorticoids exposure. M2c macrophages are involved in inflammation resolution, tissue remodeling, and secretion of anti-inflammatory cytokines such as CCL13, CCL16, and CCL18. M2d are activated by IL-6 and adenosine signaling and secrete high levels of TGF-β and CCL18. They promote angiogenesis and suppress inflammation and T cell proliferation (Strizova et al. 2023; Singer et al. 2025).

Macrophages present in TME are usually termed tumor-associated macrophages (TAMs). Although they originate mostly from circulating monocytes, part of them come from tissue resident macrophages (Bied et al. 2023). TAMs differ in their origin and phenotype depending on the type of tumor. Despite their high plasticity and phenotypic diversity, TAMs are commonly categorized into two opposite polarization states – M1 and M2 phenotypes (de Visser and Joyce 2023). Usually, high infiltration of M2-like TAMs is associated with worse outcomes and poor overall survival in many tumors (Bruni et al. 2020). Their pro-tumor effect results from the secretion of immunosuppressive, pro-angiogenic, and pro-metastatic chemokines, such as TGF-β, IL-10, VEGF, and MMPs (Gonzalez et al. 2018; Gao et al. 2022). The influence of M1-like TAMs is bidirectional – through secreting proinflammatory cytokine, e.g., CXCL9/10/11, TNF, INF-γ, IL-1, or IL-6, they promote a pro-inflammatory environment, cytotoxic T-cells recruitment, and anti-tumor response which correlates with better prognosis in several cancers (Cho et al. 2018). On the other hand, it has been demonstrated that M1-like TAMs in some cancers promote malignant tumor metastasis (Cho et al. 2018; Xiao et al. 2018). Furthermore, macrophages may shape the pre-metastatic niche to create a hospitable environment for tumor cells and the development of secondary tumors.

Although M1/M2 classification is still widely used, it is now considered overly simplistic and insufficient to capture the extensive heterogeneity of macrophages. Single-cell RNA sequencing (scRNA-seq) has enabled a more comprehensive characterization of TAMs’ phenotypes and subsets. A recent pan-cancer analysis identified several major TAM subpopulations, including resting and activated C1QC+ TAMs, ISG15+ TAMs, SPP1+ TAMs, and THBS1+ myeloid-derived suppressor cells (MDSCs). Notably, all of these populations, except resting C1QC+ macrophages, were absent in adjacent healthy tissues (Nguyen et al. 2026). The authors proposed that C1QC+ TAMs originate from tissue-resident macrophages, whereas ISG15+ and SPP1+ TAMs are likely derived from circulating blood monocytes. Functionally, C1QC+ TAMs appear to support tissue homeostasis and maintain tissue integrity during the early stages of tumor development. These macrophages are also associated with improved responses to immune checkpoint inhibitor therapy. In contrast, blood-derived THBS1 + MDSC–SPP1+ TAMs infiltrate the TME during later stages of cancer progression and contribute to a more aggressive tumor phenotype through the establishment of an immunosuppressive environment, promotion of angiogenesis and epithelial-mesenchymal transition (EMT), and a poorer response to checkpoint inhibitors. In addition to these pan-cancer TAM populations, several cancer-type-specific TAM subsets have also been identified. These include TREM2+ TAMs in colorectal, lung, breast, hepatic, and renal cancers, as well as melanoma; SIGLEC1 + TAMs in breast cancer; SIGLEC10+ TAMs in hepatic cancer; MARCO+ TAMs in prostate and lung cancer and glioblastoma; FOLR2+ TAMs in breast and lung cancer; and APOC1+ TAMs in colorectal cancer and glioblastoma. Importantly, the gene expression signatures of these subpopulations vary across different tumor types, highlighting the context-dependent nature of TAM phenotypes (Rakina et al. 2024). Furthermore, transcriptomic analyses have not only enabled the identification of TAM subpopulations across different types of cancers but also led to the identification of tumor-specific TAM markers (Table 1) (Yang et al. 2024).

Chemotherapy is a widely recognized form of cancer treatment targeting rapidly growing and dividing cancer cells. The goals of chemotherapy can be curative, palliative, or adjunctive – increasing the efficacy of other treatments such as radiotherapy or surgery. Most cancer drugs are derived from natural sources such as plants and bacteria, while others were obtained from synthetic or semisynthetic processes. Usually, a combination of differently acting chemotherapeutics is used rather than monotherapy, in order to maximize their cytotoxic effect on cancer cells and minimize the risk of developing resistance. Chemotherapeutics include a wide variety of cytotoxic drugs with different modes of action that target critical processes for cell proliferation. Most of them interfere with DNA, RNA, or protein synthesis during the S or M phase of the cancer cell cycle. The most important and most commonly used types include alkylating agents (e.g., cyclophosphamide, ifosfamide), anthracyclines (e.g., doxorubicin), taxanes (e.g., paclitaxel), inhibitors of topoisomerases (e.g., irinotecan, etoposide), kinase inhibitors (e.g., bortezomib), nucleotide analogs (e.g., azathioprine, fluorouracil (5-FU), gemcitabine), platinum-based agents (e.g., cisplatin) and *vinca* alkaloids (e.g., vinblastine, vincristine). Due to their mechanisms of action, most chemotherapy drugs show activity not only in cancer cells, but also in physiologically frequently multiplying cells such as those in bone marrow, gastrointestinal tract, and hair follicles, thus common toxicities associated with such agents include myelosuppression (often also resulting in immunosuppression), mucositis, nausea, vomiting, diarrhea, alopecia, fatigue, sterility, infertility, and infusion reactions (Hossain and Haldar Neer 2023; Anand et al. 2023). Unfortunately, little is known about the direct effects of chemotherapeutic agents on tumor-associated macrophages. Therefore, in this review, we discuss the interplay between commonly used chemotherapeutics and tumor-associated macrophages, recognizing that this relationship is bidirectional. Specifically, chemotherapeutics can alter TAMs’ phenotype and function, and macrophages, in turn, can influence tumor behavior.

**Table 1:** Macrophage markers across tumor types. Based on Yang et al. (2024)

	CD68	CD163	ISG15	SPP1	MARCO	C1q	CD47	CD206	PD-1/PD-L1	TREM2	GPNMB	LAIR1	Tim-3	CD204	CCL18	CCL20	CD38	CXCL9	CXCL10	FABP4	FOLR2	HIF-1α	IL-10	SLC40A1
Nervous System																								
Glioblastoma	+	+	+	+	+																			
Glioma	**+**	**+**	**+**	**+**	**+**						**+**			**+**										

Head & Neck																								
Oral squamous tumor cells	**+**	**+**	**+**	**+**								**+**												

Respiratory system																								
Lung cancer	**+**	**+**	**+**	**+**	**+**			**+**	**+**			**+**												
Non-small cell lung cancer	**+**	**+**	**+**	**+**	**+**			**+**					**+**		**+**									

Digestive system																								
Gastric cancer	**+**	**+**	**+**	**+**			**+**		**+**				**+**											
HBV-associated hepatocellular carcinoma	**+**	**+**	**+**	**+**								**+**												
Hepatocellular carcinoma	**+**	**+**	**+**	**+**	**+**						**+**													**+**
Intrahepatic cholangiocarcinoma	**+**	**+**	**+**	**+**			**+**																	
Pancreatic cancer	**+**	**+**	**+**	**+**		**+**	**+**	**+**			**+**					**+**								
Pancreatic ductal adenocarcinoma	**+**	**+**	**+**	**+**	**+**																			
Colon cancer	**+**	**+**	**+**	**+**									**+**											
Colorectal cancer	**+**	**+**	**+**	**+**		**+**		**+**																

Urinary system																								
Clear cell renal cell carcinoma	**+**	**+**	**+**	**+**		**+**																		
Renal cancer	**+**	**+**	**+**	**+**						**+**												**+**		
Renal carcinoma	**+**	**+**	**+**	**+**		**+**																		
Muscle-invasive bladder cancer	**+**	**+**	**+**	**+**					**+**														**+**	

Female reproductive system																								
Breast cancer	**+**	**+**	**+**	**+**			**+**		**+**	**+**				**+**			**+**	**+**	**+**		**+**			
Ovarian cancer	**+**	**+**	**+**	**+**						**+**														

Musculoskeletal System																								
Osteosarcoma	**+**	**+**	**+**	**+**																**+**				
Sarcoma	**+**	**+**	**+**	**+**						**+**														

## Drug-specific effects on macrophage-tumor dynamics

### Fluorouracil

5-fluorouracil (5-FU) is an antimetabolite chemotherapeutic developed in 1957 by Heidelberger. It is extensively used in many malignant tumors, such as gastric, colorectal, breast, and pancreatic. Its mechanism of action involves inhibition of thymidylate synthase, as well as incorporation into DNA and RNA, and as a result, disruption of those molecules (Vodenkova et al. 2020). Despite its central role as a first-line therapeutic in many cancers, its low bioavailability, high rate of degradation, chemoresistance development (Patras et al. 2016), and gastrointestinal toxicity (Abdellateif et al. 2020) are its limitations in clinical application. Among immune cells present in TME, TAMs are key promoters of tumor growth and might mediate tumor response to chemotherapy (Wei et al. 2019).

*In vitro* studies indicate that TAMs exert a complex and context-dependent influence on the responsiveness of cancer cells to 5-FU. In colorectal cancer (CRC), TAMs were shown to increase chemosensitivity of C26 colon cancer cells to 5-FU by attenuating inflammatory and angiogenic factors, such as M-CSF, IL-1α, IL-1β, VEGF, or bFGF. On the contrary, TAMs were also shown to protect cancer cells against 5-FU-induced oxidative stress by maintaining ROS levels in the physiological range of CRC cells (Patras et al. 2016). Importantly, TAMs not only exert a protective effect on colon cancer cells against 5-FU but also actively contribute to the development of chemoresistance to 5-FU in these cells. A key factor implicated in this process is secreted by THP-1 or hPBMC-derived TAMs, chemokine CCL22 (Wei et al. 2019; Zhang et al. 2023b), which, through the PI3K/AKT axis, upregulates the expression of GRP78, which interacts and translocates MRP1 transporter to the tumor cells, increasing 5-FU efflux and resistance (Zhang et al. 2023b). Moreover, 5-FU-primed mouse BMDM (bone marrow-derived macrophages) contribute to chemoresistance in CRC cells through the secretion of putrescine, a polyamine that suppresses death of CRC cells by attenuating JNK-caspase-3 pathway-mediated apoptosis (Zhang et al. 2016), and simultaneously promotes tumor cell proliferation (Farriol et al. 2001). Similar mechanisms have also been described in gastric cancer (GC), where bidirectional crosstalk between macrophages and tumor cells contributes to therapeutic failure. While M2 macrophages promote chemoresistance, 5-FU-resistant GC cells can, in turn, induce macrophage polarization towards M2 phenotype. TAMs further reinforce resistance and decrease apoptosis of GC cells. Mechanistically, the chemokine CXCL5 has been identified as responsible for TAMs-induced resistance, which, via activation of PI3K/AKT/mTOR pathway, promotes 5-FU resistance and suppresses apoptosis of GC cells (Su et al. 2022). Additionally, GC cells treatment with 5-FU leads to ROS accumulation, which induces activation and stabilization of hypoxia-induced factor 1α (HIF-1α), and, as a result, through expression of HMGB1 (one of the major damage-associated molecular patterns) leads to macrophage recruitment and polarization towards M2 phenotype. TAMs, in turn, secrete GDF15, which enhances chemoresistance and provides positive feedback for ROS production and sustainment of HIF-1α (Yu et al. 2020a).

*In vivo* studies further support the role of TAMs in promoting resistance to 5-FU-based chemotherapy. Increased TAM infiltration and elevated HMGB1 expression were observed in gastric cancer xenograft models following 5-FU treatment, confirming the involvement of macrophage recruitment during therapy-induced stress responses (Yu et al. 2020a). Similarly, immunohistochemical analyses of tumors from the CRC mouse model demonstrated that 5-FU administration significantly increased infiltration of CD68+ TAMs. Importantly, depletion of macrophages using clodronate liposomes (CL) markedly reduced TAM abundance in xenograft tumors irrespective of 5-FU treatment, further supporting the contribution of macrophages to the tumor response to chemotherapy (Zhang et al. 2016).

Clinical data are consistent with observations from experimental models. In patients with CRC treated with the FOLFOX4 regimen, high infiltration of CD206+ TAMs together with increased GRP78 expression correlated with disease relapse and poor responsiveness to 5-FU-based chemotherapy (Zhang et al. 2023b). Similar observations were reported in GC, where elevated infiltration of CD68+ macrophages was associated with resistance to 5-FU treatment (Su et al. 2022). Furthermore, macrophage phenotype represents a critical factor in therapeutic efficacy. While M1 macrophages’ presence has been shown to enhance the effectiveness of 5-FU, M2 macrophages are associated with therapeutic failure and development of drug resistance (Dong et al. 2022).

### Doxorubicin

Doxorubicin (Dox) is a potent chemotherapeutic drug, used for the treatment of multiple cancers, both solid and hematologic. Dox belongs to the anthracycline group and owes its anticancer properties to its ability to intercalate into DNA, inhibit topoisomerase II, induce oxidative damage and apoptosis (Kciuk et al. 2023). It is also known to induce caspase-dependent immunogenic cell death (Casares et al. 2005) and exposure of calreticulin – an “eat me” signal for dendritic cells (Obeid et al. 2007). Despite its widespread application, it has been proven to cause toxicity, which may lead to failure of therapy (Sonowal et al. 2021). Besides toxicity, patients often suffer from pain, fatigue, sleep disturbance, and loss of appetite. Such symptoms are similar to those seen in response to sickness, which are driven by IL-1β.

Indeed, in *in vitro* studies, Dox increased expression of pro-IL-1β and induced secretion of mature IL-1β in LPS-primed mouse BMDM in ASC, caspase-1, and NLRP3-dependent manner (Sauter et al. 2011). IL-1β is a pro-inflammatory cytokine that has been shown to play a critical role in many physiological events. It mediates inflammatory response, prostaglandin production, and activation of B-cells and T-cells, but also promotes angiogenesis (Caronni et al. 2023). And as it has been shown by Zhang et al. (2018a), IL-1β produced by THP-1-derived TAMs induces EMT in hepatocellular carcinoma (HCC). Furthermore, TAMs also influence the efficacy of doxorubicin treatment. It has been shown that the presence of TAMs decreased the sensitivity of triple-negative breast cancer (TNBC) cell lines to Dox treatment in 2D and 3D conditions. It can be explained by the TAMs’ secretion of IL-10, which activates IL-10R/STAT3/Bcl-2 pathway (Emami et al. 2021), and IL-6, which, through c-Jun, upregulates GSTP-1 – a phase II detoxifying enzyme, that regulates resistance and is overexpressed in drug-resistant breast cancers (Dong et al. 2020). Moreover, M2 extracellular vesicles contain miR-223, which has been shown to contribute to the development of Dox resistance by downregulating FBXW7, a crucial tumor suppressor protein (Gao et al. 2020).

*In vivo* studies further confirmed enhanced IL-1β-associated inflammatory responses following Dox treatment. In WT mice exposed to Dox, increased circulating levels of IL-1β, TNF, IL-6, G-CSF, CXCL1, CXCL10, and CCL2 were detected in peripheral blood. In contrast, IL-1R1-deficient mice exhibited elevated levels of IL-6, G-CSF, CXCL1, CXCL10, and CCL2, whereas induction of IL-1β and TNF was attenuated. Notably, the concentrations of all analyzed proinflammatory cytokines were markedly lower in IL-1R1 knockout mice compared with WT animals, highlighting the central role of IL-1β signaling in Dox-induced systemic inflammation (Sauter et al. 2011). Similarly, IL-1β-secreting TAMs were identified in an in vivo mouse model of pancreatic ductal adenocarcinoma (PDAC). The study demonstrated that these macrophages originated from tumor-infiltrating monocytes recruited in response to PGE2 and TNF produced by PDAC cells. In turn, IL-1β-producing TAMs promoted inflammatory reprogramming of tumor cells and enhanced their pathogenic properties, including further production of PGE2 and TNF, thereby establishing a proinflammatory positive feedback loop within the tumor microenvironment. (Caronni et al. 2023). However, IL-1β-mediated inflammation prevents metastasis-initiating cell colonization and reduces metastasis in the breast cancer mouse model (Castaño et al. 2018), which indicates a complex and context-dependent role of this cytokine. A recent study in a murine glioblastoma model demonstrated that the addition of Dox to standard radiotherapy (RT) and temozolomide (TMZ) did not alter overall macrophage infiltration within the TME, but significantly reduced the proportion of M2-like TAMs (CD206+) and CSF1R expression, whereas RT + TMZ alone had no such effect. Moreover, Dox increased the production of pro-inflammatory cytokines, including IL-1β, IL-6, IL-12, IL-23, and TNF, in BMDMs from treated mice, consistent with in vitro findings. Mechanistically, Dox binds to lactate dehydrogenase A (LDHA) in TAMs, enhancing glycolysis and lactate production. This lactate accumulation subsequently promotes the transcription and hypersecretion of M1-associated pro-inflammatory cytokines (Liu et al. 2026).

### Paclitaxel

Paclitaxel (PTX) was first approved for ovarian cancer therapy in 1992, and since then, it has been widely used in the treatment of many other tumors. It has a unique mechanism of action – PTX promotes microtubule assembly and prevents microtubule dissociation, therefore blocks cell cycle progression and mitosis (Zhu and Chen 2019). Although PTX is widely used in cancer therapy, its specific effects on tumor-associated macrophages have not yet been fully elucidated.

*In vitro* studies on THP-1 and human monocyte-derived macrophages demonstrate that PTX induces M1-like phenotype in a TLR4-dependent manner. Notably, PTX reverses the M2-polarizing effects induced by gastric cancer cells in co-culture (Yamaguchi et al. 2017). Further studies on mouse BMDM macrophages with different TLR4 expression profiles confirm that PTX promotes macrophage differentiation towards M1-like phenotype through the TLR4 pathway, and impairs polarization towards M2 phenotype (Wanderley et al. 2018). This shift is accompanied by upregulation of MHC II, CD86, and TLR2 and downregulation of CSF1R, leading to enhanced Antibody-Dependent Cellular Phagocytosis (ADCP). The crucial role of CSFR1 in the phagocytic capacity of macrophages was confirmed by siRNA downregulation, which resulted in enhanced phagocytic capacity. This suggests PTX may act as a universal adjuvant for anticancer antibody therapies (Cao et al. 2022). Consistently, PTX treatment of mouse LPS-primed BMDM stimulated with ATP or nigericin promotes NLRP3 inflammasome activation by promoting assembly of NLRP3 with protein containing caspase recruitment domain (ASC). By increasing α-tubulin acetylation, PTX accelerates the trafficking and assembly of NLRP3 with ASC, subsequently increasing IL-1β secretion and pyroptosis (Zeng et al. 2019). Recent evidence highlights the long non-coding RNA LINC00337 as a key driver of tumor progression. In breast cancer, it enhances the secretion of pro-tumorigenic factors (IL-13, CCL2, and M-CSF), which induce M2-like macrophage polarization. Silencing LINC00337 reduces these levels and diminishes the viability and migration of cancer cells. Interestingly, PTX treatment downregulates LINC00337, suggesting that its therapeutic efficacy partially stems from disrupting the LINC00337-mediated tumor-macrophage crosstalk. (Xing et al. 2021).

*In vivo* experiments in breast cancer mouse models confirm the link between LINC00337 and PTX. PTX treatment effectively counteracts the oncogenic effects of LINC00337, inhibiting tumor growth and normalizing the elevated levels of IL-13 and CCL2. Furthermore, PTX administration reduces the expression of the M2 marker CD163 in TAMs (Xing et al. 2021). While PTX may not alter the total frequency of TAMs in the tumor microenvironment, it significantly reduces M2 macrophages (F4/80+/CD206+) subpopulation. Correspondingly, PTX reduced CD206+ macrophage amount in melanoma-bearing mice, whereas this effect was diminished in TLR4-deficient mice, confirming repolarization of macrophages in TLR4-dependend manner (Wanderley et al. 2018). While PTX-mediated TLR4 activation generally promotes a tumor-suppressive M1 phenotype, the resulting inflammatory response can be multifaceted. In murine models of breast and lung carcinoma, PTX administration has been shown to induce IL-1β production within splenic macrophages. Notably, plasma derived from these PTX-treated mice enhanced the invasive capacity of cancer cells in vitro. While the systemic depletion of IL-1β in bone marrow-derived cells slightly improved the primary therapeutic efficacy of PTX, it paradoxically led to an increased burden of metastatic lesions. These findings suggest that while IL-1β may limit PTX effectiveness in the primary tumor, it simultaneously plays a critical role in restraining metastatic dissemination (Voloshin et al. 2015). Given the systemic complexities associated with broad cytokine activation, focus has shifted toward site-specific modulation of the tumor microenvironment. Parayath et al. (2019) demonstrated that nanoparticles loaded with miR-125b effectively reprogrammed the immune microenvironment by promoting macrophage repolarization toward an immune-activating phenotype. In ovarian cancer-bearing mice, treatment with these particles reduced the total peritoneal macrophage population while significantly decreasing the M2/M1 (CD206/CD80) ratio. This shift was further evidenced by the upregulation of iNOS expression in these macrophages. Consequently, this targeted shift in the macrophage polarization state significantly enhanced the antitumor efficacy of PTX.

Clinical analyses of breast cancer patients have revealed that PTX treatment significantly increases the density of tumor-infiltrating macrophages compared to untreated cohorts. Genomic profiling further identified three PTX-associated genes-IFT46, PEX11A, and TMEM23-whose expression correlates negatively with macrophage abundance and serves as a predictor of favorable survival outcomes. These findings suggest that PTX modulates the immune landscape, potentially establishing a regulatory feedback loop where macrophage infiltration influences the expression of drug-response genes within the breast cancer microenvironment (Shen et al. 2020). Single-cell RNA sequencing and spatial transcriptomics data from TNBC cohorts show that, similarly to in vitro findings, paclitaxel (PTX) exerts a key immunomodulatory effect by activating TLR4 signaling in tumor-associated macrophages (TAMs). This reprograms TAMs toward enhanced antigen cross-presentation and phagocytosis, promoting CD8+ T-cell antitumor responses and improving synergy with PD-1/PD-L1 blockade therapies (Choi et al. 2024).

### Gemcitabine

Gemcitabine (GEM) was first introduced in the late 1990s as a therapeutic agent for pancreatic ductal adenocarcinoma. Gemcitabine is an antimetabolite whose anticancer activity primarily arises from its incorporation into DNA by DNA polymerases, leading to premature chain termination and consequent cell-cycle arrest (Beutel and Halbrook 2023). For many years, GEM has been used as a first-line chemotherapy agent for various types of cancers, yet resistance to this treatment remains a great challenge.

*In vitro* studies by Buchholz et al. (2020) demonstrated that conditioned medium (CM) derived from GEM-pretreated THP-1 cells or murine BMDMs reduced the toxicity of the GEM treatment. This observation was further supported by analyses of GEM uptake in THP-1 cells and BMDM, which revealed that these cells metabolize the native form of GEM and accumulate its active form. Collectively, these observations suggest that TAMs might act as drug scavengers in TME. Another mechanism by which macrophages promote gemcitabine resistance involves the release of extracellular vesicles (EVs). EVs isolated from M2 macrophages derived from murine peritoneal cells were shown to decrease the sensitivity of pancreatic cancer cells to GEM. miR-222-3p was identified as a key mediator of the EV-induced effects in cancer cells, acting through suppression of TSC1 and subsequent activation of the oncogenic PI3K/AKT/mTOR signaling pathway, ultimately enhancing chemoresistance (Guo et al. 2023). Notably, CM derived from GEM-treated pancreatic cancer cells increased the migration, invasiveness, and proliferation of murine macrophages compared with CM from untreated cancer cells. Moreover, GEM-treated CM promoted macrophage polarization toward the M2 phenotype, suggesting that GEM exposure alters the expression of immunomodulatory cytokines in cancer cells. Indeed, increased expression of IL-8 and CCL2 was observed. Neutralization of IL-8 in GEM-treated CM attenuated its effects on macrophage migration, invasiveness, and proliferation, although it did not affect M2 polarization (Deshmukh et al. 2018). Furthermore, M2 macrophages release deoxycytidine (dC), a nucleoside that inhibits GEM activity through molecular competition (Halbrook et al. 2019). Spek et al. (2022) identified that the key transcription factor involved in dC biosynthesis in macrophages is C/EBPδ. Therefore, combining HDAC (histone deacetylase) inhibitors – suberoylanilide hydroxamic acid (SAHA) and trichostatin A (TSA), which have been shown to downregulate C/EBPδ (Ullmann et al. 2021), with gemcitabine may be a strategy to improve the efficacy of the cancer treatment.

*In vivo* studies on mouse models confirm the critical role of TME in modulating the response to GEM. Pharmacological depletion of macrophages in a pancreatic cancer mouse model enhanced gemcitabine effectiveness compared to when macrophages were present (Buchholz et al. 2020). Moreover, studies on the PDAC mouse model showed that soluble VCAM-1 (sVCAM-1), present within pancreatic tumor tissue, promotes resistance to GEM by enhancing macrophage infiltration and increasing the proportion of M2-polarized macrophages. Accordingly, treatment combining GEM with an anti-sVCAM-1 antibody reduced tumor growth, while PDAC allografts with silenced VCAM-1 exhibited decreased infiltration of M2 macrophages (Takahashi et al. 2020). Consistently, scRNA-seq analyses revealed that GEM treatment of the mouse PDAC model decreased the abundance of immune and non-immune cells, such as B-cells, fibroblasts, or stellate cells, but not macrophages in TME. Notably, the proportion of tissue-resident proliferating and inflammatory macrophages increased, suggesting their resistance to chemotherapy. Indeed, further experiments confirmed their inability to internalize GEM (Zhang et al. 2023a).

In clinical data, chemotherapy treatment (with nab-paclitaxel and GEM or FOLFIRINOX) also increased the percentage of TAMs (CD45+ CD11b+CD68+), while decreasing MDSCs. Consistent with observations from mouse models, the proportion of resident proliferating macrophages (CD68+ C1QC+) was also elevated following treatment. These macrophages appear to survive chemotherapy through downregulation of deoxycytidine kinase (dCK), the key enzyme responsible for GEM phosphorylation and activation, as well as through increased production of dC. In addition, they contribute to fibrosis and tissue remodeling, as indicated by high expression of matrix-remodeling genes, including SPP1 and MMP12 (Zhang et al. 2023a). Accordingly, clinical data from the PDAC cohort demonstrate a correlation between macrophage density and therapeutic outcome. Patients who underwent surgery followed by GEM treatment showed improved responses when the TAMs burden was low (Halbrook et al. 2019). Consistent with the proposed role of VCAM-1 in macrophage infiltration and GEM resistance observed *in vivo*, clinical studies showed that PDAC patients with decreased plasma VCAM-1 levels during GEM treatment exhibited longer progression-free survival and overall survival. Baseline VCAM-1 levels also tended to correlate with treatment outcome, further supporting its potential role in modulating response to GEM (Takahashi et al. 2020).

### Cisplatin

Cisplatin (CDDP) was the first metal-based anticancer drug introduced in 1978 into clinical use (Dasari and Bernard Tchounwou 2014). It is the first-in-class and is still widely used today for the treatment of various types of solid tumors, such as ovarian, testicular, and colorectal carcinomas, sarcomas, and lymphomas (Ghosh 2019). However, due to its effectiveness and frequent inclusion in therapy, resistance to this drug in tumors has become one of the major clinical problems (Makovec 2019). The basis of cisplatin’s action is the creation of cross-links in the DNA structure, which results in inhibition of acid replication and transcription and ultimately leads to cell death (Wang and Lippard 2005).

*In vitro* studies show CDDP interacts with the tumor microenvironment and TAMs in complex ways that influence cancer progression and treatment response across various cancers. It promotes the polarization of TAMs towards the M1 phenotype by enhancing the production of pro-inflammatory cytokines such as IFN-γ, TNF, IL-6, and IL-8 that recruit M1-like TAMs to tumor sites (Li et al. 2025). CDDP also alters the expression of scavenger receptors and pro-angiogenic factors like STAB1 and VEGFA present on TAMs (Larionova et al. 2023).

Several *in vivo* experiments show different effects of CDDP treatment on TAMs across various models. CDDP-containing regimens do not significantly reduce M2-like TAM (CD68+, CD206+, stabilin-1+) numbers, suggesting persistent post-treatment TAM populations that may contribute to residual disease or chemoresistance, though changes in their function/phenotype remain possible (Huang et al. 2024). It has been reported that M1 macrophages can deliver CDDP to tumor cells via exosomes, intensifying the drug’s apoptotic effects (Li et al. 2023b). However, TAMs can also modulate cancer cell nucleotide excision repair (NER) pathways after CDDP treatment, contributing to CDDP resistance (Duan et al. 2020). Some TAM-secreted cytokines, like CCL22, through pathways involving DGKα/NOX4, also increase CDDP resistance by inhibiting apoptosis in cancer cells (Chen et al. 2024). Another notable pathway activated during CDDP treatment is the adenosine A2A receptor (Ado/A2AR) signaling that activates PKA/mTOR pathways in TAMs, leading to their metabolic reprogramming, thereby creating an immunosuppressive niche in cross-resistant non-small cell lung cancer in a murine model. Moreover, CDDP increases mitochondrial biogenesis and oxygen consumption in both tumor cells and TAMs, leading to oxidative stress and elevated ROS levels (Ding et al. 2025). In breast cancer, CDDP treatment induces a mixed inflammatory activation state, with some stimulation of pro-inflammatory markers while maintaining M2-like TAM populations, suggesting partial polarization towards anti-tumor M1 phenotypes without extensive total TAM population depletion (Larionova et al. 2023). CDDP-containing regimens do not significantly reduce M2-like TAM (CD68+, CD206+, stabilin-1+) numbers, suggesting persistent post-treatment TAM populations that may contribute to residual disease or chemoresistance, though changes in their function/phenotype remain possible (Huang et al. 2024). In ovarian cancer, TAMs contribute to CDDP resistance by promoting translesion DNA synthesis (TLS) in cancer cells, mediated by an upregulation of DNA polymerase eta (Pol η) (Chatterjee et al. 2025). Due to TLS, CDDP-damaged DNA templates can still be accurately replicated, thus promoting and ensuring tumor cell survival despite treatment. TAMs in this context predominantly exhibit an alternatively activated M2 phenotype (CD206+, CD163+) and are localized in invasive tumor margins, supporting tumor progression, cancer stemness, and drug resistance. In a study concerning glioblastoma, it has been shown that CDDP reduces M2-like TAM-mediated immunosuppression by downregulating the CD47-SIRPα signaling pathway, thereby promoting macrophage-mediated phagocytosis of tumor cells, enhancing chemo- and immunotherapy sensitivity (Li et al. 2025). Lung cancer cells secrete factors (e.g., MCP-1, PGE2) that recruit and maintain M2-like TAMs, which antagonize CDDP efficacy (Kawaguchi et al. 2023). Depleting these M2 TAMs enhances CDDP’s antitumor effects.

### Cyclophosphamide

Cyclophosphamide (CP, CTX) is the most widely used alkylating agent of modern times, introduced in 1959 and used as treatment in a variety of both solid and hematopoietic/lymphoid tissue tumors (Emadi et al. 2009).

In *in vitro* studies, TAMs phenotypes after CP treatment show notable changes primarily characterized by a decrease in pro-tumor M2-like macrophages and a shift toward more anti-tumor M1-like phenotypes (Genard et al. 2017). The NF-κB signaling pathway is crucial for inflammatory responses and chronic inflammation associated with tumor progression. CP can modulate NF-κB signaling in TAMs, impacting their secretion of cytokines and capacity to foster tumor progression or resistance (Mandracci et al. 2025).

*In vivo*, in several models including lung carcinoma and breast cancer, an initial enrichment of M2-skewed TAMs characterized by markers such as MRC1 (CD206), Tie2, and VEGF-A, soon after CP treatment was introduced (Hughes et al. 2015). These M2-like TAMs can potentially be involved in tumor relapse and angiogenesis shortly after chemotherapy but represent a dynamic state rather than a fixed phenotype. Although direct studies on TAMs’ shifts are limited, related myeloid changes have been observed where CP increased certain monocyte populations expressing MHC II within the tumors, consistent with differentiation toward immature dendritic cells or macrophages (Leong et al. 2019). This could suggest that CP induces a shift in TAMs’ precursors, potentially favoring anti-tumor phenotypes. Additionally, CP augments tumor-specific CD8+ T cell cytolytic function, which indirectly influences TAMs’ activation and polarization toward pro-inflammatory M1-like states (Gephart et al. 2025). CP can impact the colony-stimulating factor 1 (CSF-1) and its receptor (CSF-1R) axis, a major pathway regulating TAMs’ polarization (Shang et al. 2025). CSF-1R signaling activates downstream pathways such as PI3K/AKT/FOXO1, ERK1/2, and STAT3 that promote TAMs’ polarization toward the M2 phenotype, which generally support tumor progression. CP downregulates and inhibits this pathway and thus reduces M2 polarization and tumor-supportive TAMs’ functions. CP also increases the number of Fcγ receptors (such as FcγRIII and FcγRIV) on macrophages while downregulating inhibitory receptors (e.g., FcγRIIB), enhancing macrophage ADCP, which synergizes with antibody therapies in resistant tumor microenvironments, including bone marrow niches (Roghanian et al. 2019). In multiple myeloma, CP promotes macrophage migration to tumors and skews TAMs towards a pro-inflammatory phenotype by upregulating chemoattractant receptors CCR2/CCR5 and activating Fcγ receptors, which enhance ADCP. Studies concerning breast cancer generally report an increase in M1-like macrophage phenotype shift and their infiltration into the tumor after CP treatment. However, in murine models of breast cancer, CP induced perivascular TAMs to express increased VEGF-A and the angiopoietin receptor Tie2 levels, which in turn promoted tumor revascularization and regrowth after chemotherapy. It was suggested that blocking CXCR4 signaling can prevent this rebound effect (Hughes et al. 2015). Studies on combined cisplatin and CP treatment showed no significant decrease in total TAM numbers (CD68+, CD206+) post-chemotherapy, thus indicating that CP-based regimens may not significantly reduce TAMs’ abundance but could still alter TAMs’ function or phenotype (Larionova et al. 2020). In lung cancer, CP promotes polarization of TAMs from M2 to M1 phenotypes, increasing production of pro-inflammatory cytokines like TNF and IFN-γ and fostering tumoricidal activity (Hao and Chen 2025). In melanoma, CP has been associated with promoting chronic inflammation-dependent immunosuppression, which can hinder anti-tumor immune responses (Sevko et al. 2013). In glioblastoma, CP treatment reduced immunosuppressive TAM populations and enhanced pro-inflammatory macrophages, thus potentially boosting treatment efficacy (Pennisi et al. 2025). It has been reported that CP treatment can create a microenvironment favorable for prostate cancer skeletal metastasis by causing an expansion of myeloid lineage cells, including TAM precursors, in bone marrow and peripheral blood (Park et al. 2012). This was associated with an increased production of cytokines like CCL2, IL-6, and VEGF-A, which promote myelogenesis and macrophage recruitment. This suggests that CP can indirectly influence TAM populations to foster metastasis in this type of cancer. In solid tumors, like pancreatic and ovarian cancers, CP-induced TAMs shift from M2-like phenotypes to M1-like states, aiding therapeutic efficacy (Wang et al. 2024). In murine sarcoma and lymphoma models, TAMs surviving CP treatment stimulated the proliferation of residual tumor cells, potentially contributing to tumor relapse (Milas et al. 1994; Laurent et al. 2024).

### Vincristine

Vincristine (VCR) is a vinca alkaloid, widely used in chemotherapy. It is a cytostatic agent whose antineoplastic activity results from its binding to tubulin, thereby inhibiting mitosis. Despite strong anticancer effects, its biological activity is unspecified, which results in various side effects, such as nausea, vomiting, diarrhea, hair loss, and, most severe, neurotoxicity (Škubník et al. 2021). VCR is well known to exhibit immune-modulatory effects. Its cytotoxic activity results in the release of intracellular substances and inflammatory mediators from dead and injured cells (Kiguchi et al. 2009).

Early *in vitro* research suggests that VCR may inhibit Antibody-Dependent Cellular Cytotoxicity (ADCC) through the secretion of reactive oxygen intermediates (O_2_^−^ and H_2_O_2_) by macrophages (Gudewicz 1988). Although subsequent research has primarily focused on the effects of VCR on macrophages in the context of pain and allodynia, macrophage-derived factors induced by VCR may also influence tumor progression and antitumor immunity. VCR has been shown to suppress M2-like polarization of PBMC-derived macrophages through inhibition of the JAK2/STAT3 signaling pathway. In addition, VCR-treated M2 macrophages exhibited reduced expression of PD-L1, CCL17, and CXCL10, while CXCL9 expression was increased (Fujimura et al. 2018). Similarly, Gao et al. (2021) demonstrated that VCR promotes polarization of RAW264.7 macrophages toward the M1 phenotype through activation of the ERK1/2 and p38-MAPK pathways.

The immunomodulatory effects of VCR have also been confirmed *in vivo*. In C57BL/6J mice, VCR treatment increased the levels of IL-1β, IL-6, and TNF within the longitudinal muscle myenteric plexus. Importantly, macrophage depletion prior to VCR administration reduced the expression of these cytokines, highlighting the central role of macrophages in the VCR-mediated inflammatory response (Gao et al. 2021). In a mouse model of allodynia, VCR also increased the infiltration of peripheral macrophages into the sciatic nerve and lumbar dorsal root ganglion. These macrophages expressed high levels of IL-6, a key mediator of vincristine-induced mechanical allodynia (Kiguchi et al. 2008), but in the tumor context, IL-6 usually correlates with better survival prognosis (Cho et al. 2018). In melanoma mouse models, VCR reduced the proportion of M2 macrophages (CD206 + CD163+) within the TME. Moreover, macrophages isolated from the TME of VCR-treated mice and cultured in vitro showed decreased secretion of CCL22 (Fujimura et al. 2018). Since CCL22 promotes cancer invasion, metastasis, and recruitment of regulatory T cells to the tumor site (Chen et al. 2022), its reduced expression may contribute to more pro-inflammatory and anticancer responses. Given the tumoricidal properties of M1 macrophages, several therapeutic strategies have been proposed to modulate macrophage polarization in combination with VCR treatment. For example, simultaneous administration of VCR and IFN-γ, or sequential treatment with VCR followed by IFN-γ, significantly reduced neuroblastoma growth in mouse models. In contrast, administration of IFN-γ before VCR attenuated the pro-inflammatory effects, highlighting the importance of treatment scheduling (Zeki et al. 2023). Another approach to modulating macrophage function involves direct targeting of tumor-associated macrophages via sialic acid receptors, as demonstrated by sialic acid-modified vincristine sulfate liposomes, which disrupt the immunosuppressive tumor microenvironment and activate persistent antitumor immunity (Yan et al. 2023).

### Etoposide

Etoposide (ETO, ETP) was introduced in 1971 and has since been widely used in the treatment of solid tumors and lymphomas. As a topoisomerase II inhibitor, etoposide stabilizes the transient DNA–topoisomerase II complex, leading to accumulation of double-strand breaks and subsequent cell death (Meresse et al. 2004). Beyond its direct cytotoxic activity, etoposide exhibits immunomodulatory properties, and etoposide-based regimens have been investigated in clinical studies for the treatment of macrophage activation syndrome (MAS) (He et al. 2022). Accumulating evidence indicates that macrophages can modulate tumor cell responses to etoposide in a phenotype-dependent manner.

*In vitro* studies demonstrated that murine BMDMs reduced the levels of phosphorylated histone H2AX (γH2AX), a marker of DNA double-strand breaks, in tumor cells co-cultured and treated with ETO. Moreover, co-cultures of BMDMs and tumor cells exposed to ETO exhibited a lower proportion of cells with sub-2 C DNA content compared to tumor cell monocultures, indicating reduced apoptosis and diminished drug efficacy (Olson et al. 2017). Furthermore, THP-1-derived macrophages co-cultured with ETO-treated cancer cells exhibited increased IL-8 expression, while prolonged exposure of cancer cells to ETO enhanced their phagocytic uptake by macrophages (Kawagishi et al. 2001). IL-8 appears to play an important role in ETO sensitivity, as tumor-derived IL-8 has been shown to activate NF-κB-dependent anti-apoptotic signaling pathways, thereby reducing cellular sensitivity to etoposide (Maxwell et al. 2007). Importantly, the effects of THP-1-derived macrophages on ETO response in co-culture with cancer cells depend on their polarization state. M1 macrophages enhance tumor cell sensitivity to ETO, whereas M2 macrophages exert a pronounced protective effect by markedly reducing ETO-induced apoptosis. Notably, ETO treatment does not substantially alter the overall transcriptional identity of macrophages in vitro, as expression profiles largely retain their original M1 or M2 signatures. Nevertheless, selective transcriptional changes have been observed, including reduced CCL18 expression in M2 macrophages and increased IL6 expression in M1 macrophages (Genin et al. 2015). In contrast, other studies have described an increase in pro-inflammatory cytokines, such as CCL17, CXCL5, and CXCL10, in ETO-treated M2 macrophages, suggesting partial acquisition of inflammatory features (Ohuchi et al. 2020). These mediators are associated with diverse tumor-promoting and antitumor functions, including recruitment of regulatory or cytotoxic T cells depending on tumor context, induction of EMT, and enhancement of cytotoxic immune responses (Ye et al. 2022; Zou et al. 2022). Consistently, THP-1-derived M0 macrophages exposed to supernatants from ETO-treated cancer cells displayed an M1-like phenotype, further supporting the notion that ETO can indirectly modulate macrophage polarization through tumor-derived signals (Malekghasemi et al. 2020). In parallel, ETO-stimulated human bone marrow stromal cells (hBMSCs), particularly in the presence of G-CSF, secrete IL-8, which has been further shown to activate the IL-8/CXCR2/mTOR/c-MYC signaling axis and promote proliferation and expansion of human hematopoietic stem cells (hHSCs) as well as their differentiation into progenitor cells. However, ETO also induces a significant proportion of hBMSCs to undergo cell-cycle arrest in the G0/G1 phase, indicating long-term stromal toxicity (Kang et al. 2020) Although enhanced proliferation of hHSCs may exert suppressive effects in certain leukemic contexts (Li et al. 2023a), IL-8 signaling has been broadly associated with increased tumor cell survival, migration, angiogenesis, and resistance to chemotherapy across multiple cancer types, and high IL-8 expression correlates with adverse outcomes in preclinical and clinical studies (Fousek et al. 2021).

In an *in vivo* mouse model of lymphocytic leukemia, injection of ETO-treated leukemic cells resulted in elevated levels of macrophage inflammatory protein-2 (MIP-2), the murine homolog of IL-8, in peritoneal exudate cells. Consistent with in vitro observations, increased MIP-2 levels in mouse model correlated with enhanced phagocytosis of cancer cells by macrophages Similarly, in a mouse model of peripheral blood hematopoietic stem cell (PB-HSC) mobilization, combined treatment with ETO and G-CSF tended to induce higher circulating levels of murine IL-8 homologs, including MIP-2, KC, and LIX, compared with cyclophosphamide plus G-CSF treatment, although no significant differences were observed relative to the G-CSF-only group. Importantly, hematopoietic progenitor cells (HPCs) isolated from the peripheral blood of mice treated with ETO and G-CSF generated significantly higher numbers of colony-forming units (CFUs), including granulocyte, erythrocyte, monocyte, megakaryocyte, and macrophage colonies, compared with the G-CSF-only group. Interestingly, expression of MMPs associated with HSC mobilization did not differ substantially in peripheral blood between treatment groups. However, in the bone marrow of mice treated with ETO and G-CSF, MMP9 expression was increased, whereas SDF-1 (CXCL12) expression was reduced compared with other groups (Kang et al. 2020). Overall, these findings indicate that ETO profoundly modulates immune responses and macrophage function; however, its effects on macrophage polarization and antitumor immunity appear to be highly context-dependent and remain incompletely resolved.

## Macrophages as drug carriers

**Fig. 2:**
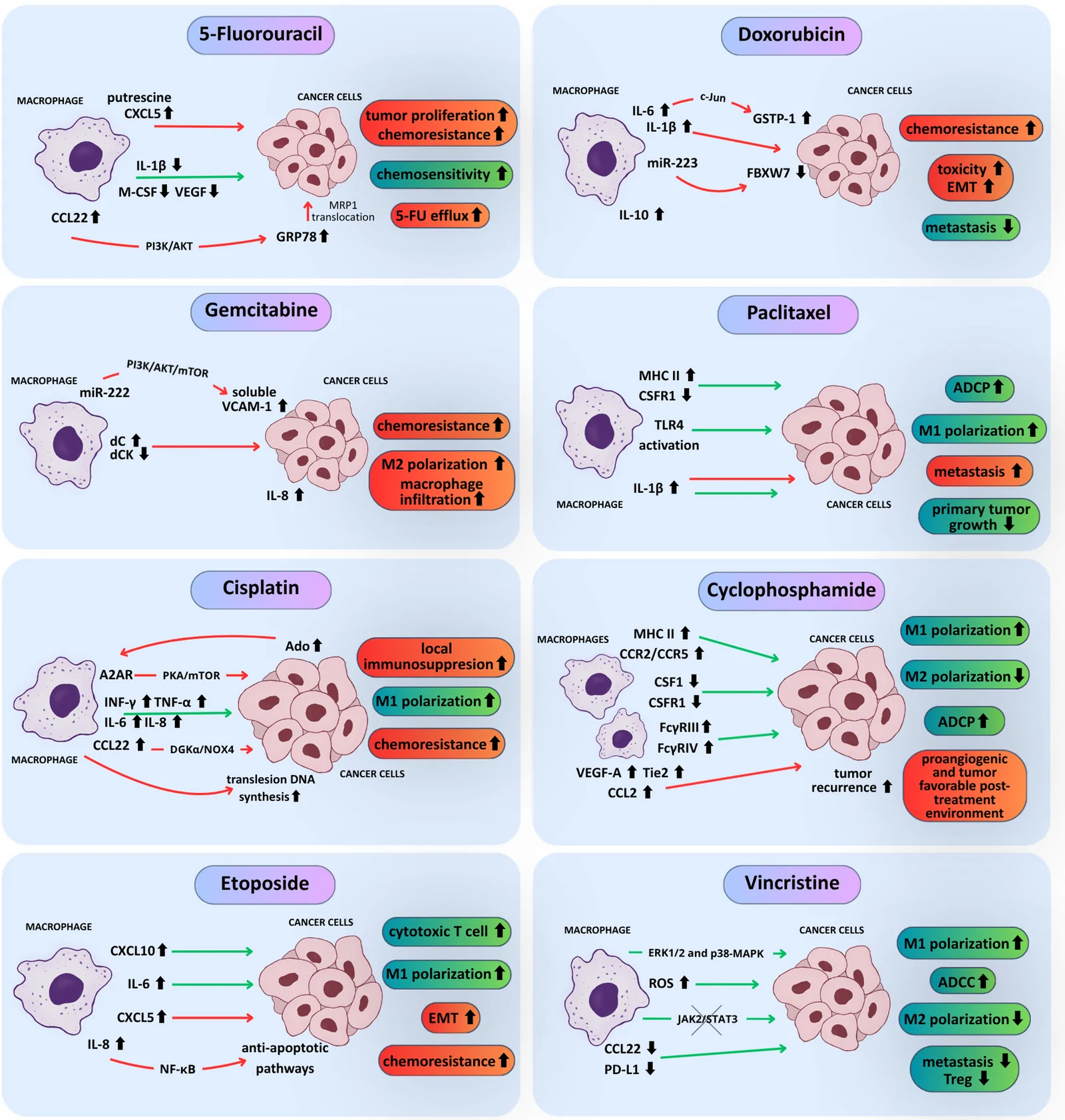
Macrophages and macrophage-derivatives as drug carriers

Given that macrophages, both tissue resident and monocyte-derived, play a pivotal role in modulating tumor behavior and are highly abundant within the tumor microenvironment, there is increasing interest not only in targeting TAMs directly, but also in exploiting their intrinsic chemotactic properties and tumor-homing properties for drug delivery (Fig. 2).

One such strategy employed silica nanocapsules (SNs) designed to encapsulate large amounts of Dox within macrophages. These nanocapsules (DSN) consist of a drug-silica nanocomplex core surrounded by a solid silica shell, which confers resistance to oxidation and degradation within macrophage phagolysosomes, thereby enabling efficient transport of the drug to tumor tissue prior to its local release. Intravenous administration of DSN-bearing macrophages into tumor-bearing mice resulted in macrophage migration to tumors and localized Dox release, leading to effective tumor suppression (Zhang et al. 2018b).

An alternative approach utilized poly(lactic-co-glycolic acid) (PLGA) nanoparticles loaded with Dox and surface-modified with mannose to selectively target TAMs, which overexpress the mannose receptor. In this system, Dox was encapsulated in PEGylated acid-sensitive, sheddable polyethylene glycol-coated mannose-modified PLGA nanoparticles (AS-M-PLGA-NPs). Following administration into tumor-bearing mice, the nanoparticles bound to macrophages via mannose receptor-mediated interactions. This strategy reduced macrophage abundance within the TME and suppressed tumor growth compared to non-coated nanoparticles and Dox alone (Niu et al. 2016).

More recently, a “backpack” strategy was developed to deliver Dox to tumors using macrophages as carriers. In this approach, Dox was encapsulated in DSPE-PEG-biotin-modified liposomes, which were tethered to the surface of streptavidin (DSPE-PEG-STA) modified macrophages through streptavidin–biotin interactions. This design avoided the potential cytotoxicity associated with intracellular drug loading. Following administration into tumor-bearing mice, macrophages carrying Dox-loaded liposomes migrated to tumors and released the drug locally. This delivery system prolonged survival and reduced systemic toxicity compared with conventional treatment approaches (Yang et al. 2022).

In another strategy, exosomes derived from M1 macrophages were used as delivery vehicles for PTX. Exosomes isolated from macrophage culture media were loaded with PTX and subsequently administered to tumor-bearing mice. PTX-loaded exosomes efficiently infiltrated tumor tissue and were internalized by cancer cells. In addition to enhancing the production of pro-inflammatory cytokines by macrophages in vitro, these exosomes exhibited greater antitumor efficacy *in vivo* compared with PTX alone (Wang et al. 2019).

Similarly, PTX-loaded albumin nanoparticles coated with macrophage plasma membranes demonstrated enhanced cellular uptake, increased tumor accumulation, and superior antitumor activity compared with uncoated nanoparticles or free PTX. In this approach, macrophage membranes were isolated and used to coat PTX-containing albumin nanoparticles (PTX-ANPs). After administration into tumor-bearing mice, the membrane-coated nanoparticles preferentially accumulated within tumors and were internalized by cancer cells, resulting in improved therapeutic efficacy and more selective tumor targeting (Cao et al. 2020).

## Discussion and conclusions

**Fig. 3:**
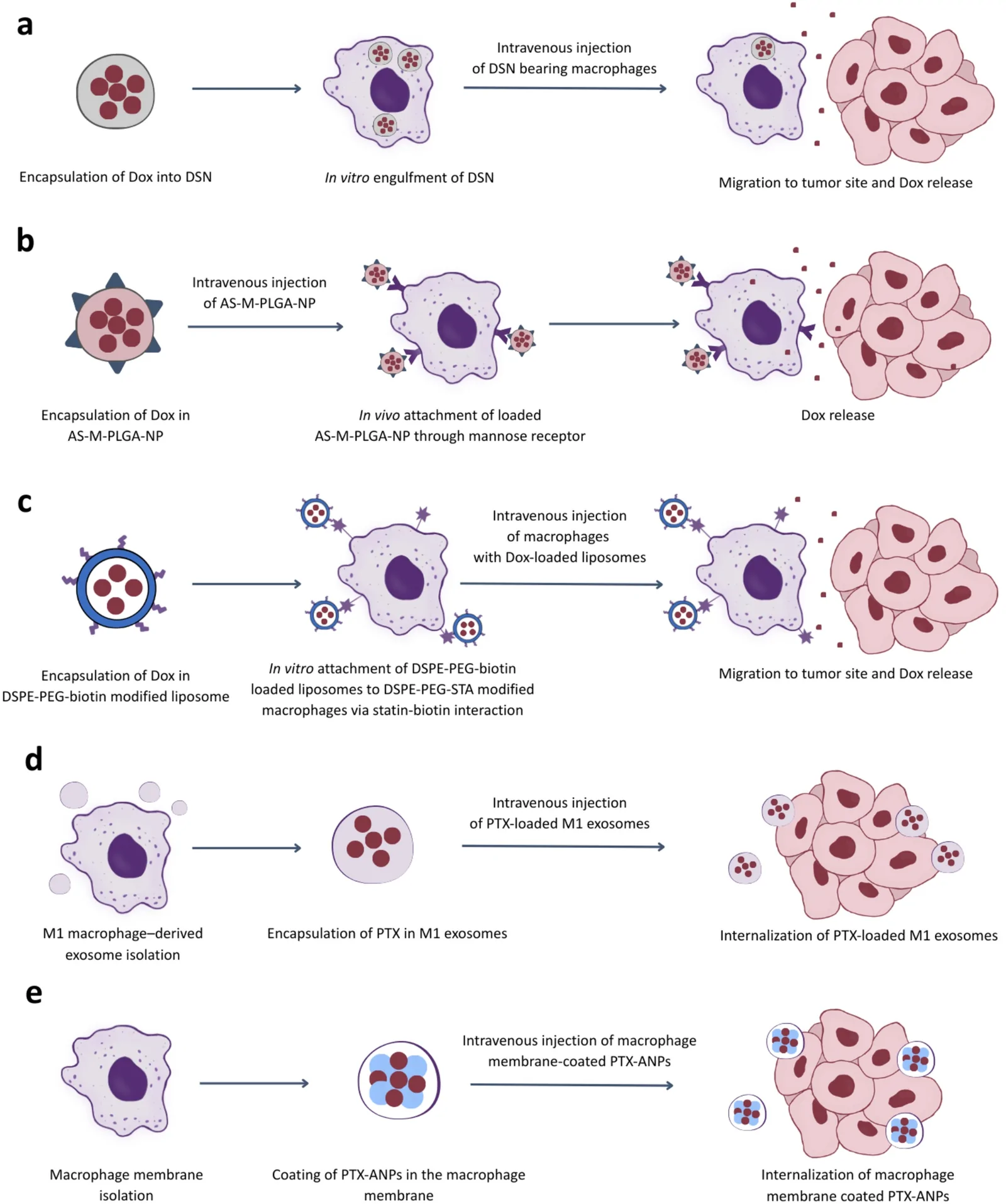
Macrophage-cancer interactions upon drug treatment. Green indicates antitumor effects, whereas red indicates protumor effects. Arrows labeled with pathway names indicate activation of specific signaling pathways, while crossed arrows indicate pathway inhibition

When it comes to clinical relevance, evidence gathered and analyzed in this work underlines the importance of TAMs’ phenotype modulation in strategies aimed at improving clinical outcomes of chemotherapy. The interaction between chemotherapeutic agents and macrophages is bidirectional; while therapeutic agents can alter the phenotype and function of TAMs, these macrophages, in turn, significantly shape the tumor’s response to chemotherapeutic treatment and overall behavior (Fig. 3). Although the chemotherapeutic agents discussed belong to different classes and exhibit distinct mechanisms of action, all of them affect the TME, including TAMs. Importantly, these effects are highly context-dependent and influenced by factors such as experimental design, drug-specific mechanisms, cancer type, and stage of tumor progression, making it difficult to determine whether a given chemotherapeutic exerts an overall beneficial or detrimental effect on the TME.

The presence of M2-like TAMs has been associated with reduced efficacy of chemotherapy and the development of drug resistance through multiple mechanisms, including the secretion of IL-8 (GEM, ETO) and CCL22 (5-FU, CDDP) (Kawagishi et al. 2001; Deshmukh et al. 2018; Wei et al. 2019; Kang et al. 2020; Chen et al. 2022; Zhang et al. 2023b). Several agents, including DOX, PTX, CDDP, CP, VCR, and ETO, have been reported to suppress M2-like polarization and induce a shift toward a more pro-inflammatory, M1-like phenotype (Fujimura et al. 2018; Wanderley et al. 2018; Roghanian et al. 2019; Ohuchi et al. 2020; Liu et al. 2026). However, this phenotypic transition is often accompanied by increased secretion of IL-1β, which, in certain contexts, may promote EMT (Voloshin et al. 2015; Zhang et al. 2018a) or decrease the effectiveness of chemotherapy. Furthermore, some therapeutics affects CSF-1/CSF1R axis – CTX, GEM, and PTX downregulate it, which shifts macrophages towards M1-like phenotype, but also increase ADCP (Roghanian et al. 2019; Cao et al. 2020; Liu et al. 2026).

It is important to emphasize that chemotherapy may not only alter macrophage phenotype but also affect their recruitment and proliferative capacity. For example, 5-FU increases recruitment of macrophages towards the tumor site (Yu et al. 2020b), and ETO promotes proliferation of HPCs (Kang et al. 2020). In PDAC tissue, proliferating macrophages are present, and cancer-associated fibroblast-derived CSF-1 promotes their proliferation through p21 induction. Chemotherapy further increases the proportion of p21-high macrophages (Zuo et al. 2023). Consistently, PDAC patients treated with nab-paclitaxel and GEM or FOLFIRINOX exhibited a higher percentage of proliferating macrophages (Zhang et al. 2023a).

Given that macrophages strongly influence tumor responses to chemotherapy and that the presence of M2-like TAMs is closely associated with chemoresistance, strategies aimed at reprogramming macrophage phenotype or depleting macrophage populations appear highly promising. For instance, the simultaneous administration of IFN-γ with VCR significantly enhanced its therapeutic efficacy (Zeki et al. 2023), while macrophage depletion improved the effectiveness of GEM treatment (Buchholz et al. 2020). Furthermore, since the presence of M1-like macrophages has been shown to enhance the efficacy of immune checkpoint inhibitors (ICIs) (Choo et al. 2018; Yu et al. 2024), there is a premise for combining chemotherapeutic agents that promote TAM polarization toward an M1-like phenotype with ICIs, such as anti-PD-L1 therapies.

Given the dual origin of TAMs – either from tissue-resident macrophages or from circulating blood monocytes (Nguyen et al. 2026) – the site of phenotypic modulation is not always straightforward to pinpoint. Although many studies have demonstrated altered macrophage infiltration or phenotype following chemotherapy, the cellular origin of these macrophages often remains unclear. Evidence indicates that chemotherapy can act at multiple biological levels, beginning in the bone marrow (BM), continuing through peripheral circulation, and culminating within TME. Several findings point to systemic, BM-level effects. Cytotoxic agents such as CP or CDDP induce extensive immunogenic tumor cell death and systemic hematological stress, thereby activating emergency myelopoiesis in the BM. This response promotes the expansion of hematopoietic stem and progenitor cells (HSPCs) and myeloid lineage populations through cytokine-mediated signaling involving factors such as CCL2, IL-6, and VEGF-A (Park et al. 2012). Furthermore, Dox has been reported to alter the BM niche in mice, leading to a more pro-inflammatory phenotype in BMDMs (Liu et al. 2026), while ETO modified MMP9 and SDF‑1 expression in the BM but not in peripheral blood (Kang et al. 2020). During circulation, monocytes carrying epigenetic and metabolic imprints respond to chemoattractant gradients generated by the treated tumor through chemokine receptors such as CCR2 or CCR5, guiding their trafficking toward peripheral tissues. Finally, upon arrival at the tumor site, the TME determines the ultimate polarization state of TAMs. Local chemotherapy‑induced conditions (including hypoxia, ROS accumulation, and tumor‑derived exosomes) shape macrophage differentiation and functional specialization (Zhang et al. 2026). Consequently, the BM acts as the initiator of myeloid cell priming, whereas the TME functions as the final regulator shaping TAM phenotype toward either pro-inflammatory M1-like or immunosuppressive M2-like states.

Tumor-associated macrophages (TAMs) play a pivotal role in the progression and metastasis of cancer. Their diverse origins, plasticity, and ability to polarize into distinct M1 and M2 subtypes highlight their complexity and the significant impact they have on tumor biology. As our understanding of TAMs’ biology continues to evolve, their potential as both prognostic biomarkers and therapeutic targets will likely play an increasingly important role in cancer treatment strategies (Salmaninejad et al. 2026).

## Data Availability

No datasets were generated or analysed during the current study.
